# Sevoflurane reverses cisplatin resistance in neuroblastoma cells through the linc00473/miR-490-5p/AKT1 axis

**DOI:** 10.15537/smj.2022.43.11.20220549

**Published:** 2022-11

**Authors:** Huiqing Li, Xiaobo Fu, Huiyu Guo, Yue Sun, Di Wang, Zengzhen Zhang

**Affiliations:** *From the Department of Anesthesiology (Li, Fu, Guo, Sun, Zhang), Shandong Provincial Third Hospital, Jinan, Shandong; and from the Department of Clinical Experiment (Wang), The Eighth Medical Center of Chinese PLA General Hospital, Beijing, China.*

**Keywords:** sevoflurane, cisplatin-resistant tumors, lncRNA, ceRNA

## Abstract

**Objectives:**

To determine whether sevoflurane regulates cisplatin resistance in neuroblastoma cells.

**Methods:**

The SH-SY5Y cell line with cisplatin-resistant phenotype (SH-SY5Y-SR) was generated. Cells were co-treated with sevoflurane and cisplatin to seek the sevoflurane function on cisplatin resistance. Key targets of sevoflurane treatment were determined using sequencing (ribonucleic acid [RNA-seq]). Cells were then transfected with specific vectors. Linc00473 and microRNA-490-5p (miR-490-5p) levels were detected using reverse transcriptase quantitative real-time reverse transcription PCR (RT-qPCR). Linc00473-miR-490-5p binding was confirmed using a luciferase reporter-gene assay. After treatment, cell proliferation, viability, and caspase-3 activity were measured to determine the effects of treatment on tumor cells. Each experimental result is based on three independent experiments.

**Results:**

Co-treatment with sevoflurane and cisplatin markedly improved the sensitivity of SH-SY5Y-SR cells to cisplatin, which inhibited the occurrence of cisplatin resistance. The RNA-sequencing analysis and RT-qPCR showed that sevoflurane inhibited linc00473 expression. Overexpression of linc00473 promoted cell proliferation, inhibited apoptosis, and promoted cisplatin resistance. The linc00473/miR-490-5p/V-akt murine thymoma viral oncogene homolog 1 (AKT1) axis was found to mediate the regulatory effects of sevoflurane on cisplatin resistance.

**Conclusion:**

Sevoflurane has great clinical potential against cisplatin-resistant tumors. Further animal experiments and clinical trials are required to achieve this goal.

**P**rimary intracranial neuroblastoma is a highly malignant tumor. Nearly half of all neuroblastoma cases occur in infants under 2 years of age. Neuroblastoma accounts for approximately 6-10% of tumors in children, and approximately 15% of children with neuroblastoma succumb to the disease.^1^

Cisplatin is one of the most widely used drugs for neuroblastoma. Its mechanism of action is to dissociate chlorine and crosslink it with the deoxyribonucleic acid (DNA) of tumor cells, thereby, destroying the DNA.^2^ In recent years, the increasing prevalence of cisplatin-resistant cases has affected the use of this drug.^3^ There are several reasons for cisplatin resistance, including decreased cisplatin accumulation, increased glutathione and metallothionein levels, and enhanced DNA repair. The expression of oncogenes and changes in signal transduction pathways during apoptosis are also associated with cisplatin resistance.^3-4^

Sevoflurane, a commonly used anesthetic, can reportedly regulate tumor development.^5^ Preliminary molecular mechanism studies have demonstrated that sevoflurane regulates classic tumor signal path, such as the Wingless and int-1 (WNT)/β-catenin pathway and phosphatidylinositol-4,5-bisphosphate 3-kinase (PI3K)/AKT signaling.^5^ However, whether sevoflurane can regulate cisplatin resistance and the related molecular mechanisms remain unclear.

Here, we observed that 5% sevoflurane pre-treatment of cisplatin-resistant neuroblastoma cells could markedly reverse cisplatin resistance, and this makes it convenient for us to explore the molecular mechanism. A large body of evidence has confirmed that long non-coding ribonucleic acids (lncRNAs) and microRNAs (miRNAs) are key factors in tumor development and drugs resistance.^6,7^ Several lncRNAs and miRNAs reportedly affect the mechanisms of action of sevoflurane.^8,9^ In this study, using RNA-sequencing (RNA-seq), we observed that linc00473 is a potential target of sevoflurane. Linc00473 is a recently discovered lncRNA that can induce many types of cancer.^6^ In addition, the function of linc00473 in chemotherapeutic drug resistance has been discovered.^7^

This study sought to clarify the principle behind linc00473-mediated sevoflurane reversal of cisplatin resistance.

## Methods

This experimental study was carried out from January 2020 to June 2022 at Shandong Provincial Third Hospital, Jinan, China. The ethics committee of our hospital had proved that this work does not require ethical approval.We used the Web of Science website to search for prior related publications.^10^

### Cell culture and generation of cisplatin-resistant cells

A cell line derived from human neuroblastoma, SH-SY5Y (we termed it SH-SY5Y-NC), was cultured at 37°C and 5% carbon dioxide (CO_2_). According to previously reported methods, 11 cisplatin-resistant SH-SY5Y cells (designated as SH-SY5Y-SR) were generated. Cells in good condition were used for the subsequent experiment, while the cells with poor status or mycoplasma infection were excluded.

### Sevoflurane-treated cells

After the cells were inoculated into a culture plate for 24 hours (h), they were placed in a closed plexiglass box. The air inlet of this plexiglass box was connected to an anesthesia vaporizer, and the air outlet was connected to a gas analyzer. A gas content ratio of 5% CO_2_, 21% oxygen (O_2_), and 74% nitrogen (N_2_) was initially maintained. Before the experiment, sevoflurane gas was delivered to the plexiglass box at 3 L/min with an anesthetic vaporizer, and sevoflurane content was monitored using a gas analyzer. When the concentration of sevoflurane was 5%, the air inlet and outlet were closed, and the cells were treated in the closed plexiglass box for 3 h and then placed in an incubator for 24 h, followed by the corresponding detection analyses. Control cells were not exposed to sevoflurane gas, and the other culture conditions were the same as those in the experimental group.

### 3-(4,5-dimethylthiazol-2-yl)-2,5-diphenyl-2H-tetrazolium bromide (MMT) assay

An MTT kit (ab211091, abcam, Cambridge, UK) was used to measure cell viability in 96-well plates. Briefly, cells were subjected to specific treatments, and then were incubated with MTT for 3 hours. Subsequently, dimethyl sulfoxide (DMSO) was joined for fifteen minutes, and optical density (OD) values were recorded at 570 nanometer (nm) and converted to the quantitative value of cells based on a standard curve.

### Apoptosis assay

An Annexin V Staining Kit (APOAF-20TST, Sigma-Aldrich, St. Louis, MO), combined with flow cytometry analysis, was used to examine apoptosis. A caspase-3 substrate was used to measure the fluorescence intensity of caspase to determine the activity of caspase-3, which reflects the level of apoptosis.

### Reverse transcriptase quantitative real-time reverse transcription PCR (RT-qPCR) detection

Using TRIzol (15596026, Thermo Fisher Scientific, Waltham, MA), we purified RNAs from the cells to be tested. We used one Step RT-PCR Kit (QYR0604, qualityard biotechnology Co., Ltd, Beijing, China) to perform the RT-qPCR on lncRNA, and used GAPDH as an internal control for data normalization. We used TaqMan miRNA One-Step Analysis Kit (4427975, Thermo Fisher Scientific, Waltham, MA) to perform the RT-qPCR on miR-490-5p, and used U6 as an internal control for data normalization.

### Plasmid construction and transfection

Human linc00473 complementary DNA (cDNA) was cloned into the plasmid cDNA3.1 (pcDNA3.1) (designated pcDNA3.1-linc00473). The linc00473 siRNA and a non-targeting control (NC), as well as a mimic and inhibitor of miR-490-5p, were obtained from Fitgene (Guangzhou, China). Lipofectamine 2000 (12566014, Thermo Fisher Scientific, Waltham, MA) was used to transfect the cells.

### Luciferase reporter-gene activity

Constructed wild-type or binding-site mutant plasmids of linc00473 were transfected, together with specific miR-490-5p mimics or inhibitors. Luciferase activity was detected according to the instruction.

### Statistical analysis

Each experimental result is based on three independent experiments. IBM SPSS Statistics for Windows, v.24 (IBM Corp., Armonk, N.Y., USA), was used to conduct a 2-tailed Student’s t-test. If the calculation result was *p*<0.05, the difference was considered significant.

## Results

### Generation and phenotype of cisplatin-resistant neuroblastoma cells

**Figure 1:**
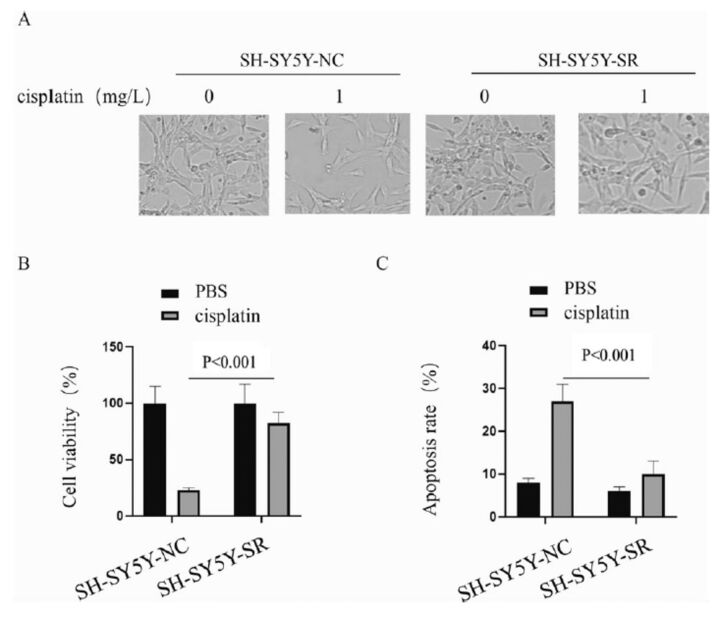
- Identification of cisplatin resistant neuroblastoma cell line. SH-SY5Y-NC and SH-SY5Y-SR cells were treated with 0.1mg/l cisplatin for 24 hours, and then the cell viability and apoptosis rate were determined. A) The morphology of SH-SY5Y-NC and SH-SY5Y-SR cells after certain treatments; B) The cell viability (%) of SH-SY5Y-NC and SH-SY5Y-SR cells after after certain treatments were determined; C) The apoptosis rate (%) of SH-SY5Y-NC and SH-SY5Y-SR cells after after certain treatments were determined.

First, we characterized the constructed SH-SY5Y-SR cells. The results revealed that cisplatin (0.1 mg/L) could inhibit cell viability with statistical significance against SH-SY5Y-NC, while the inhibition rate of SH-SY5Y-SR cells was approximately 15% (Figure 1 A&B); at the same time, cisplatin at the indicated concentrations could significantly promote SH-SY5Y-NC cell apoptosis, whereas this function was significantly weakened in SY5Y-SR cells (Figure 1C).

### Sevoflurane reverses cisplatin-resistant phenotype and decreases linc00473 expression in SH-SY5Y-SR cells

**Figure 2:**
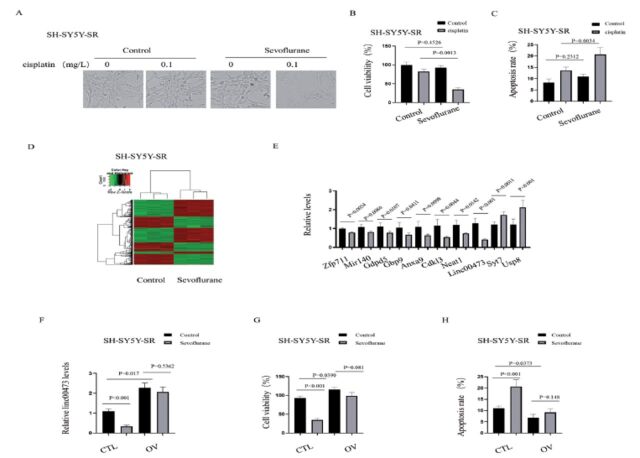
- Sevoflurane reverses the cisplatin-resistant phenotype and decreases linc00473 expressions in SH-SY5Y-SR cells. A-C) SH-SY5Y-SR cells were pretreated with 5% sevoflurane and then treated with 0.1mg/l cisplatin for 24 hours. A) The morphology of SH-SY5Y-SR cells after different treatments; B) The cell viability (%) of SH-SY5Y-SR cells after different treatments were determined; C) The apoptosis rate (%) of SH-SY5Y-SR cells after different treatments were determined; D) SH-SY5Y-SR cells were treated with 5% sevoflurane and then cultured for 24 hours. RNA-seq was used to analyze the global differences in transcripts between sevoflurane treated group and control group. E-G) SH-SY5Y-SR cells were transfected with linc00473 overexpression plasmid (OV) or control plasmid (CTL) for 24 hours, and then pretreated with 5% sevoflurane and then treated with 0.1mg/l cisplatin for 24 hours. E) The relative linc00473 levels were determined by qRT-PCR assay after different treatments. F) The cell viability (%) of SH-SY5Y-SR cells after different treatments were determined; G) The apoptosis rate (%) of SH-SY5Y-SR cells after different treatments were determined.

We observed that 5% sevoflurane could partly reverse the cisplatin-resistant phenotype of SH-SY5Y-SR cells, as reflected by down-regulated cell viability (Figure 2 A&B) and enhancement of apoptosis (Figure 2C) after cisplatin treatment in the 5% sevoflurane pre-treated group, compared with the non-pre-treated group.

We performed RNA-seq to analyze global differences in mRNA transcripts between the 5% sevoflurane-treated SH-SY5Y-SR cells and the control group (Figure 2D). Table 1 lists the 10 transcripts with the most significant differences, of which eight targets were downregulated, while 2 were upregulated. We examined these 10 targets using RT-qPCR and observed that the expression patterns were similar to those as revealed by RNA-seq results (Figure 2E). Among them, the linc00473 transcript exhibited the greatest difference between the sevoflurane pre-treatment group and the control cohort (Figure 2E).

**Table 1:** - Top 10 differential targets from the RNA-seq results in SH-SY5Y-SR cells between sevoflurane treated group and control group.

Targets	Log_2_(fold change)	*P*-value
Linc00473	-3.21	7.91E-07
Gdpd5	-2.24	0.000114
Zfp711	-2.09	0.00021
Cdkl3	-1.94	0.000532
Mir140	-1.87	0.00072
Neat1	-1.02	0.00073
Anxa9	-1.82	0.000861
Gbp9	-1.77	0.000899
Usp8	2.33	0.001076
Syt7	2.30	0.001083

Next, we constructed a linc00473 overexpression plasmid (OV group) and transfected it into SH-SY5Y-SR cells. Predictably, 5% sevoflurane did not affect linc00473 levels in the OV group (Figure 2F). Using this model, we determined the role of linc00473 in mediating the function of sevoflurane. Interestingly, 5% sevoflurane pre-treatment, followed by cisplatin exposure, decreased cell viability and induced apoptosis, but these effects were not evident in the OV group (Figure 2 G&H). These results indicated that linc00473 mediates sevoflurane function.

### Linc00473 acts as a carcinogen in SH-SY5Y-NC cells

**Figure 3:**
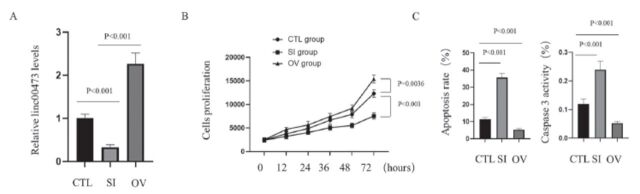
- Linc00473 is a carcinogen of SH-SY5Y-NC cells. A) SH-SY5Y-NC cells were transfected with linc00473 overexpression plasmid (OV), siRNA (SI) or control plasmid (CTL) for 24 hours, and then the relative linc00473 levels were determined by qRT-PCR assay after different treatments. B) SH-SY5Y-NC cells were transfected with linc00473 OV or siRNA (SI) for certain period, and then the cells proliferations were determined by MTT assay. C) SH-SY5Y-NC cells were transfected with linc00473 OV, siRNA (SI) or control plasmid (CTL) for 48 hours, and then the apoptosis rates (%) and casepase-3 activity were determined.

Next, we studied the effects of linc00473 on tumorigenesis. We transiently transfected the linc00473 overexpression plasmid (OV group) and the siRNA (SI group) into SH-SY5Y-NC cells (Figure 3A). The cell proliferative activity was higher in the OV group than control and lower in the SI cases (Figure 3B). The apoptosis rate and caspase-3 activity were lower in the OV group and higher in the SI cases (Figure 3C). These results revealed that linc00473 acts as a carcinogen in SH-SY5Y-NC cells.

### Linc00473 is a key factor in cisplatin resistance

**Figure 4:**
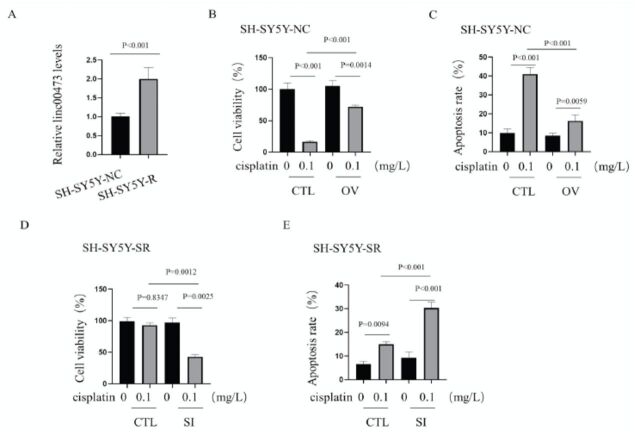
- Linc00473 is a key factor of cisplatin resistance in SH-SY5Y-SR cells. A) Comparing the relative linc00473 levels between SH-SY5Y-SNC cells and SH-SY5Y-SR cells. B-C) SH-SY5Y-NC cells were transfected with linc00473 overexpression plasmid (OV) or control plasmid (CTL) for 24 hours, and then pretreated with 5% sevoflurane and then treated with 0.1mg/l cisplatin for 24 hours. (B) The cell viability (%) after different treatments were determined; C) The apoptosis rate (%) after different treatments were determined. D-E) SH-SY5Y-SR cells were transfected with linc00473 siRNA (SI) or control plasmid (CTL) for 24 hours, and then pretreated with 5% sevoflurane and then treated with 0.1mg/l cisplatin for 24 hours, and then D) The cell viability (%) after different treatments were determined; E) The apoptosis rate (%) after different treatments were determined.

The linc00473 levels in SH-SY5Y-SR cells were significantly higher than in SH-SY5Y-NC cells, which led us to hypothesize that linc00473 may function in cisplatin resistance (Figure 4A). Therefore, SH-SY5Y-NC cells were transiently transfected with pcDNA3.1-linc00473 (OV group) or the negative control plasmid (CTL group) and then treated with 0.1 mg/L cisplatin. The results showed that in the CTL group, cisplatin at the indicated concentrations inhibited the survival of SH-SY5Y-NC cells by >80%, while in the OV group, the inhibition rate was significantly reduced to approximately 30%, and this difference was statistically significant (Figure 4B). Similarly, cisplatin (0.1 mg) significantly promoted apoptosis in the CTL group, whereas this effect was significantly inhibited in the OV group (Figure 4C). In addition, we transiently transfected linc00473 siRNA (SI group) or the negative control (CTL group) into SH-SY5Y-SR, and treated the cells with cisplatin (0.1 mg/L). The results showed that cisplatin at the indicated concentrations had a weak effect on inhibiting survival and promoting apoptosis in the CTL group, whereas cisplatin had a significantly stronger effect in the SI cases (Figure 4D–E). These results showed that linc00473 is a key factor in cisplatin resistance.

### Linc00473/miR-490-5p/AKT1 axis mediates regulatory effects of sevoflurane on cisplatin resistance

**Figure 5:**
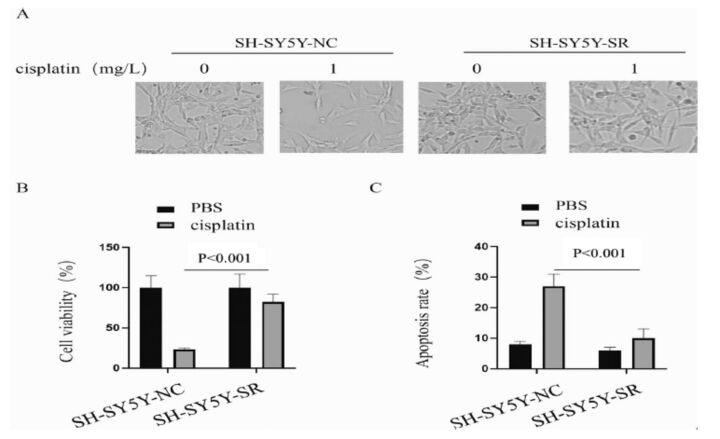
- Linc00473/miR-490-5p/AKT1 axis mediates the regulatory effect of sevoflurane on cisplatin resistance. A) Wild type and mutant linc00473 were linked with the sequence of miR-490-5p; B) Wild type and mutant linc00473 luciferase reporter, together with miR-490-5p NC, mimics and inhibitor, were co-transfected into SH-SY5Y-NC cells for 24 hours, and then the luciferase activity was determined; C-D) SH-SY5Y-NC cells were transiently transfected with the overexpression plasmid (OV), siRNA (SI) or control plasmid (CTL) of linc00473, together with miR-490-5p mimics in OV group and miR-490-5p inhibitor in SI Group and then C) proliferations were determined by MTT assay.; D) The apoptosis rate (%) after different treatments were determined. (E-F) SH-SY5Y-SR cells were transfected with indicated vectors for 24 hours, and then pretreated with 5% sevoflurane and then treated with 0.1mg/l cisplatin for 24 hours, and then E) The cell viability (%) after different treatments were determined; F) The apoptosis rate (%) after different treatments were determined. AKT1 OV: AKT1 overexpression plasmid.

Linc00473 could form complementary base pairing with miR-490-5p (Figure 5A). Next, we generated a wild-type linc00473 luciferase reporter-gene vector and a linc00473 luciferase reporter-gene vector containing certain binding site mutations (Figure 5A) and co-transfected them with miR-490-5p mimic, inhibitor, or NC. The luciferase activity of the wild-type linc00473 luciferase vector could be inhibited by the miR-490-5p mimic, while the mutant linc00473 luciferase reporter-gene vector did not change significantly with miR-490-5p mimic treatment (Figure 5B). We transiently transfected the linc00473 overexpression plasmid (OV group) and siRNA (SI group) together with the miR-490-5p mimic in the OV group and the miR-490-5p inhibitor in the SI cases. The effect of OV or SI on cell proliferation and apoptosis could be reversed by the miR-490-5p mimic or inhibitor, respectively (Figure 5 C&D). These results showed that linc00473 acts as a competitive endogenous RNAs (ceRNA) that targets miR-490-5p.

MiR-490-5p targets multiple important genes and regulates the AKT1 signaling pathway.^12^ As an important oncogene, AKT1 has been reported as a target of cisplatin resistance.^13^ Here, 5% sevoflurane could restore the sensitivity of SH-SY5Y-SR cells to cisplatin, but 5% sevoflurane + miR-490-5p inhibitor or 5% sevoflurane + AKT1 overexpression plasmid (AKT1-OV) weakened the ability of sevoflurane to restore cisplatin sensitivity (Figure 5 E&F), indicating that the linc00473/miR-490-5p/AKT1 axis mediates the regulation of cisplatin resistance by sevoflurane.

## Discussion

Neuroblastoma is a frequently occurring malignant tumor in children. Cisplatin is effective against various types of advanced or recurrent cancers, including neuroblastoma.^14^ Unfortunately, the prevalence of cisplatin resistance is gradually increasing in patients with neuroblastoma.^14,15^ Therefore, it is important to identify new and effective anti-cisplatin-resistance neuroblastoma drugs. Our results indicated that 5% sevoflurane pre-treatment could significantly enhance the sensitivity of SH-SY5Y-SR to cisplatin. We compared mRNA transcripts in neuroblastoma cells pre-exposed to 5% sevoflurane to those in the control group using RNA-seq, and found that linc00473, a new lncRNA, was the most differentially expressed RNA between sevoflurane-treated and non-treated groups, which may function in cisplatin-mediated sevoflurane inhibition of cisplatin resistance.

Some lncRNAs can directly induce chemotherapeutic drug resistance, including cisplatin resistance. For instance, Xu et al^16^ reported that lncRNA small nucleolar RNA host gene 1 (SNHG1) promotes cisplatin resistance by regulating the miR-338-3p/ polo-like kinase 4 (PLK4) pathway. Zhang et al^17^ demonstrated that overexpression of the lncRNA cardiac IKs opposite strand/antisense transcript 1 (KCNQ1OT1) can promote cisplatin resistance, which is closely related to the regulation of the ezrin/focal adhesion kinase (FAK)/non-receptor tyrosine kinase (SRC) pathway. At present, little is known regarding the correlation between linc00473 and cisplatin resistance. To further explore the role of linc00473, we overexpressed linc00473 in neuroblastoma cells. The results showed that linc00473 is an oncogene that promotes the proliferation of cancer cells and inhibits their apoptosis. Overexpression of linc00473 can promote cisplatin resistance, whereas its inhibition can weaken cisplatin resistance.

Recent research has revealed that lncRNAs and miRNAs can interact, and thus, affect the post-transcriptional regulation of their targets by inhibiting miRNA activity.^18^ Here, miR-490-5p was found to be directly targeted by linc00473. Many studies have shown that miR-490-5p is an important tumor blocker and plays an anti-tumor role involving many key cellular pathways.^19,20^ Li et al^11^ confirmed that miR-490-5p/AKT1 is an axis that mediates the antitumor effects of miR-490-5p. AKT1 is involved in a variety of tumor-related biological processes.^21^ Several studies have confirmed that over-activation of AKT1 is an important factor in cisplatin resistance.^22,23^ Here, we observed that the inhibitory activity of sevoflurane on cisplatin resistance in neuroblastoma was markedly reversed by miR-490-5p inhibitor or AKT1 overexpression plasmid, indicating that sevoflurane inhibits cisplatin resistance by regulating the linc00473/miR-490-5p/AKT1 axis.

Sevoflurane has recently been shown to affect the development of human cancer. It was already known that sevoflurane has many advantages over other anesthetics, such as propofol, including lower toxicity and side effects, and better anti-inflammatory action.^5^ This study has added to existing information on potential applications sevoflurane in cisplatin-resistant tumors.

### Study limitation

We only carried out preliminary cell-based experiments, and the results of animal-based experiments and clinical sample validation need to be acquired in subsequent studies.

In conclusion, these findings facilitate our understanding of the role of sevoflurane in the resistance to cisplatin in neuroblastoma and may provide an experimental basis for selecting more effective anesthetics for patients.
